# Therapy of parapneumonic empyema in children: a protocol for a scoping review of the literature

**DOI:** 10.12688/f1000research.135295.1

**Published:** 2023-11-28

**Authors:** Danilo Buonsenso, Francesca Cusenza, Lucrezia Passadore, Francesca Bonanno, Carolina Calanca, Sonia Rasmi, Francesco Mariani, Susanna Maria Roberta Esposito

**Affiliations:** 1 Department of Woman and Child Health and Public Health, Fondazione Policlinico Universitario A. Gemelli Istituto di Ricovero e Cura a Carattere Scientifico (IRCCS), Rome, Italy, Rome, Italy; 2 ] Pediatric Clinic, Department of Medicine and Surgery, University of Parma, Parma, Italy, parma, Italy

**Keywords:** Parapneumonic empyema; complicated pneumonia; children

## Abstract

**Background**: Empyema (the presence of pus in the pleural space) is a severe complication of community-acquired pneumonia and significant cause of morbidity, but, fortunately, not mortality in children. Between 0.6 and 2% of pneumonias are complicated by empyema and the three main pathogens involved are Streptococcus pneumoniae, Staphylococcus aureus and group A Streptococcus 1,2,3,4. Optimal management in children, especially the choice of antibiotics, method of administration and duration of therapy, are still matter of debate and currently, lack of strong specific recommendations. This paper displays the study protocol for a scoping review that aims to summarize the available literature on the microbiological epidemiology, the treatment options, and the outcomes of pleural empyema in pediatric population.

**Methods:** Comprehensive research combining the terms pediatric (children aged 0 to 18 years) and pleural empyema will be performed on PubMed and SCOPUS to identify all eligible studies. At first, two reviewers will screen their abstract and then their full text to detecting the articles that meet the inclusion criteria. This work will be carried out independently, everyone on a different Excel spreadsheet and each researcher will be blinded to the decision of the other researcher. When the process will be completed, in case of discordance, any disagreement will be identified and resolved through discussion or with the help, when needed, of a third author.

**Dissemination:** The findings of this review will be published in a peer-reviewed journal.

## Introduction

Parapneumonic empyema is defined by the collection of pus on the pleural surfaces, and it represents one of the most common local complications of community-acquired pneumonia (CAP) in children. ^1^^‐^^5^ It has been estimated that parapneumonic effusions develop in about 1 in 100-150 children with CAP ^6^^,^^7^ but they could be discovered in as many as 40% of hospitalised children with CAP. ^8^

CAP, are caused mainly by *Streptococcus pneumoniae* ^9^ and their incidence has shown fluctuations over time. In particular, a significant global reduction in pneumococcal disease and mortality rates has been reported after the introduction of heptavalent pneumococcal conjugate vaccine (PCV7), which covers serotypes 4, 6B, 9V, 14, 18C, 19F and 23F, into the standard childhood immunisation schedule. ^10^ In the following years, however, in the USA an increase in pneumococcal empyema, related to serotypes not covered by PCV7, has been reported. ^11^ After the replacement of the PCV7 with the PCV13, which covers also serotypes 1, 3, 5, 6A, 7F and 19 A, there has been a significant reduction in incidence and rate of hospitalisation for empyema. ^12^ The introduction of PCV13 is particularly important in consideration of the strong correlation between parapneumonic empyema and serotype 1 of pneumococcus. ^13^

Other bacteria seem to be less frequently pathogens of CAP. However, other possible bacterial pathogens of parapneumonic empyema are represented by group A Streptococcus and Staphylococcus aureus. ^13^

Clinical presentation of parapneumonic empyema is similar to that of uncomplicated CAP. The presence of an empyema should be suspected in children with prolonged fever (more or equal to 7 days) and in those who do not improve after 48-72 hours of adequate antibiotic therapy. ^9^^,^^13^ In physical examination, typically, parapneumonic empyema is characterized by decreased air entry breath and dullness to percussion. ^9^

A clinical suspect of parapneumonic empyema should be confirmed performing a chest X-ray and/or pulmonary ultrasound. Ultrasound technique has a higher sensitivity than radiograph in determining extension and nature of fluid collection and it is very useful for monitoring children with empyema, considering that it does not expose to X-rays. Thoracic CT is not considered a first line exam in order to make diagnosis of empyema, but it should be performed when it is not possible to make a clear diagnosis or when there is a suspect of malignancies (i.e. Burkitt’s lymphoma).

All cases of parapneumonic empyema should be treated with empiric intravenous antibiotic therapy, covering Streptococcus pneumoniae, Streptococcus pyogenes and Staphylococcus aureus. However, in case of large effusion (> 2 cm) or compromission of respiratory function a chest drainage is essential. ^5^^,^^14^ Chest drainage is generally performed under ultrasound guide and children should be under sedation/general anaesthesia. ^14^ Intrapleural fibrinolytics (i.e. urokinase) are particularly useful in shortening hospitalisation in cases where drainage is slow, in consideration of thick or loculated fluid. ^5^^,^^14^ Thoracic surgery should be taken in consideration in cases of failure of antibiotic therapy, chest drainage and fibrinolytics. However, guidelines are unclear about which surgical procedure is best and at which timing, as well duration of drainage or of antibiotic therapy, including optimal timing about oral shift and how these issues reflect on outcomes.

This scoping review aims to analyse the optimal antibiotic therapy, defining antibiotic molecule, route of administration and duration of antimicrobial therapy.

## Review questions

Considering the importance of a mutual consensus in the clinical management of parapneumonic empyema in children, as literature already pointed out, ^15^ the main review question will be: what is the available literature about the most appropriate antibiotic treatment for paediatric PE in terms of first-line agents choice, dose, route of administration and duration?

This review will also assess the following sub-questions:

1. Which are the most frequently reported pathogens and what is their antibiotic susceptibility profile?
2. Which outcomes and complication rates of PE are the most frequently reported in literature? Which are the most frequently reported treatments, both conservative or invasive, and which leads to improved outcomes and shorter length of stay?

## Inclusion criteria

### Participants

This review will include studies performed on children and adolescents (younger than 18 years) with a confirmed diagnosis of empyema, defined as the presence of pus within the pleural cavity. The diagnosis of empyema is established by the presence of pus, positive Gram's stain, or culture, or nucleic-acid amplification tests, in the pleural fluid. We will only include studies that have documented the microbiological aetiologies, performed antimicrobial and surgical therapies, as well outcomes (at least at time of discharge).

### Concept

The main concept of this review will be empyema in all its aspects, with a particular focus on treatment options.

### Context

Considering the severity of the disease, we will not expect to find articles involving patients not hospitalized so we will include only inpatients.

### Type of sources

This review will include both randomized controlled trials and non-randomized controlled trials. All the types of observational studies, prospective and retrospective (including case-control, cohort and cross-sectional studies, small case series or single case reports) will be included.

## Methods

### Search strategy

The search will be performed by one reviewer. We started our research in April 2023 in the following bibliographic databases: PubMed and SCOPUS. There will be date restrictions: we will search from the 1st of January 2000 to 31st of March 2023. Only articles written in English will be included. The search strategy will include a combination of the following word and their synonymous: “pediatric”, “empyema” and “treatment”. The search strategy for PubMed is available in the extended data section of this protocol; the terms used for this search will be adapted for use with other bibliographic database.

### Study selection

After the search, the studies will be exported to Rayyan. A first screen to exclude duplicates will be performed by one author.

Titles and/or abstracts of studies retrieved using the search strategy will be screened independently by two reviewers to identify studies that could be inserted in the review. Full texts of potentially eligible studies will be retrieved and independently assessed for eligibility by two reviewers. Each researcher will be blinded to the decision of the other researcher. Any disagreement between them over the eligibility of studies will be resolved through discussion and, in case of further disagreement, by discussion with a third reviewer.

All the studies that will not meet the inclusion criteria will be excluded and a table with the reason why those studies were excluded will be inserted in the final manuscript.

The results of the search will be reported in the PRISMA flow diagram.

### Data extraction

Two review authors will extract data independently, everyone on a different Excel spreadsheet. Each researcher will be blinded to the decision of the other researcher. When the process will be completed, in case of discordance, any disagreement will be identified and resolved through discussion (with a third author if necessary).

An Excel file will be used to store data. When available, extracted information will include:

1. study general features: title, author, year of publication, type of study, number of patients included in the study, geographical area where the study has been performed
2. participant general features: sample size of each group, nationality, age, socio-economic status, comorbidities
3. clinical manifestation of the condition: fever (including days), cough with mucus, dyspnoea, chest pain and others.
4. main imaging findings: type of lung involvement at chest X-Ray and/or CT scan, type of CNS involvement at CT scan or MRI, type of skin involvement evaluated by ultrasound or CT scan or MRI, heart (US or CT or MRI)
5. characteristics of eventual antimicrobial treatments performed during the empyema (length of therapy, when this has been started and which antibiotic was used)
6. adjunctive treatments performed and length of therapy during the empyema (e.g., steroids or other immunomodulatory medications)
7. surgical treatments performed and length of therapy during the empyema (e.g., drainage or thoracoscopy or surgical resection)
8. outcomes (death, survival; survival with sequelae; type of sequelae)

### Data analysis and presentation

To report our findings, we will follow Preferred Reporting Items for Systematic reviews and Meta-Analyses extension for Scoping Reviews (PRISMA-ScR) Checklist.

We will produce a narrative synthesis of the findings from the studies included in the review describing the results we have obtained and providing our opinion on their interpretation. A particular focus with a narrative synthesis will be performed for antimicrobial and surgical therapy characteristics in terms of frequency of antibiotic choice, efficacy, and duration of therapy. If after this selection will be included more than 100 records, original article and those published in the last 5 years will be preferred.

We will also use tables and charts to summarize both study characteristics and the most important clinical, diagnostics, treatments, and outcomes data.

More specifically, we will summarize our findings using different tables. The first one will include the characteristics of included studies (number of studies, study design, year of publication, characteristics of the study populations and countries where studies were conducted) and the participant general features. Then we will provide different tables or figures summarizing main data about clinical presentation, imaging characteristics, treatments performed, outcomes and predictors of empyema.

This way we hope we will be able to provide a useful document containing what is currently known of pediatric empyema with the aim of informing clinicians about the general characteristics of these conditions, focusing on risk factors and early clinical features, and guide future research projects to fill current gaps.

### Study status

Protocol has been submitted and researched launched on the different datasets. Abstract screening will start after protocol submission.

### Patient and public involvement

There was no direct patient and public involvement in this review. However, the key questions that led us implementing this research project were inspired by public discussions started by family associations in the media, highlighting the importance of better comprehension of how empyema can be recognized earlier in the disease course (before clinical conditions deteriorates and cannot be controlled anymore), or empyema may also be prevented if this complication is a consequence of a previous unrecognized and untreated lung infection.

### Strengths and limitations of this study

• A scoping review can represent the best way to report on the types of evidence that are published in a certain field and our paper will provide an overview of empyema, focusing on predictors of positive outcomes.
• A scoping review can represent the best way to examine this field to guide future research on this topic.
• To report our findings, we will follow the Preferred Reporting Items for Systematic reviews and Meta-Analyses extension for Scoping Reviews (PRISMA-ScR) Checklist to ensure methodological strength to our paper.
• Only two databases were screened, and only English paper will be considered limiting the number of papers that will be included.
• No critical appraisal neither risk of bias of the included studies will be performed, considering the exploratory role of this paper.

## Data Availability

No data are associated with your article. Open Science Framework: Therapy of parapneumonic empyema in children: protocol for a scoping review of the literature, https://doi.org/10.17605/OSF.IO/J58DR. ^16^
