# Supplementary material for: Therapy of parapneumonic empyema in children: a protocol for a scoping review of the literature
Source: F1000Res. 2024 Jun 3;12:1527. Originally published 2023 Nov 28. [Version 2] doi: 10.12688/f1000research.135295.2 (PMC11294806; doi:10.12688/f1000research.135295.2)
Supplement: Peer review history

# Reviewer report: Reviewer response for version 2

**DOI:** 10.5256/f1000research.167272.r285922

**Published:** 2024-08-01

**Authors:** Catherine A Byrnes

**Affiliations:** 1 Auckland District Health Board, Starship Children's Health, Auckland, New Zealand

Thanks - I think the changes are appropriate. 

 I think it looks good for indexing.

 I have a couple of minor English edits to make to read correctly (but if you make those changes it can go straight to indexing )

 Intro para 5 'a suspicion of malignancies' 

 Intro para 6 ''or compromising respiratory function'

 intro para 6 'including optimal timing about shifting to oral antibiotics and'

 intro para 7 'defining antibiotic of choice'

 participants 'as well as outcomes'

 study selection 'could be reviewed' (delete 'inserted')

 data extraction 'when the process is completed'

 that's it - thanks

Is the study design appropriate for the research question?

Partly

Is the rationale for, and objectives of, the study clearly described?

Yes

Are sufficient details of the methods provided to allow replication by others?

Yes

Are the datasets clearly presented in a useable and accessible format?

Yes

Reviewer Expertise:

Paediatric Respiratory disease

I confirm that I have read this submission and believe that I have an appropriate level of expertise to confirm that it is of an acceptable scientific standard.

# Reviewer report: Reviewer response for version 2

**DOI:** 10.5256/f1000research.167272.r285923

**Published:** 2024-06-27

**Authors:** King-Pui Florence Chan

**Affiliations:** 1 University of Hong Kong, Queen Mary Hospital,, Hong Kong, Hong Kong

Thank you for the amendment. The protocol was revised with appropriate response to the comments.

Is the study design appropriate for the research question?

Partly

Is the rationale for, and objectives of, the study clearly described?

Yes

Are sufficient details of the methods provided to allow replication by others?

Yes

Are the datasets clearly presented in a useable and accessible format?

Not applicable

Reviewer Expertise:

Respiratory Medicine, Respiratory infection, Epidemiology

I confirm that I have read this submission and believe that I have an appropriate level of expertise to confirm that it is of an acceptable scientific standard.

# Reviewer report: Reviewer response for version 1

**DOI:** 10.5256/f1000research.148406.r272217

**Published:** 2024-05-28

**Authors:** King-Pui Florence Chan

**Affiliations:** 1 University of Hong Kong, Queen Mary Hospital,, Hong Kong, Hong Kong

The study protocol aims to provide a literature review on children with pleural empyema. The protocol can be improved by further clarification.

 1. In the method part, I am not aware of how the bacterial results is analyzed or included. Please consider elaborating it.

 2. The severity of outcomes including intensive care unit stay can be included as analysis.

 3. Some of the pleural empyema can be caused by tuberculosis, which the antimicrobial use would be different. Please consider to address this in the protocol.

Is the study design appropriate for the research question?

Partly

Is the rationale for, and objectives of, the study clearly described?

Yes

Are sufficient details of the methods provided to allow replication by others?

Yes

Are the datasets clearly presented in a useable and accessible format?

Not applicable

Reviewer Expertise:

Respiratory Medicine, Respiratory infection, Epidemiology

I confirm that I have read this submission and believe that I have an appropriate level of expertise to confirm that it is of an acceptable scientific standard, however I have significant reservations, as outlined above.

# Response

**Published:** 2024-05-29

**Authors:** danilo buonsenso

thank you, I have responded to your main comments

# Reviewer report: Reviewer response for version 1

**DOI:** 10.5256/f1000research.148406.r245793

**Published:** 2024-05-15

**Authors:** Catherine A Byrnes

**Affiliations:** 1 Auckland District Health Board, Starship Children's Health, Auckland, New Zealand

Generally this is a good proposal – responding to queries and questions generated from patients and using Prism as the backbone which is good.

1. Not much is mentioned regarding chest drains – nil in abstract, not till the 2 ^nd^ to last paragraph in the introduction and as part of the Qs – mentioned in passing. The authors need to clarify this a bit more. Is chest drainage and/or video assisted thoroscopic surgery going to be included?
2. Articles only in English or other languages. The authors should****** define the languages of the articles they are searching in.
3. Only including patients that have an identified organism – many patients are difficult to get a positive culture from – and often the published articles have some with identified organisms and some without – yet the authors here are only including those where bacteria have been isolated – can they clarify this?
4. If greater than 100 articles – will then restrict to last 5 years – better if stuck to the whole. Either do all articles or restrict to 5 years - wouldn't go with the number being a restriction.
5. Note that although this is being presented as a protocol, the review has already started.
6. In the selected word options for search – no “pleural effusion”. The authors have given the search words they have used but have not included "pleural effusion".

The manuscript could do with some English editing - I appreciate that the authors are likely writing this in a second language. Some editing is needed to put some of the phrasing into correct grammatical English.

**Abstract:**

- “are still a matter for debate”
-  “their abstracts” → “the abstracts”
- “then their full text” → “then the full text”
- “detecting” → “determine”
- “when the process will be completed” → “ is completed”
- “with help, when needed, of a third” →  “with help of a third”

**Introduction:**

- “discovered’ → seen or recognised or discerned (para 1)
- “their” → “the” (para 2)
- “less frequently” → “less frequent” (para 3)
- “suspect” → “suspicion” (para 5)
- “radiograph” → “radiography” (para 5)
- “xray” → “radiation”

**Review Qs:**

- “as literature points out” → “as documented in the available literature”

**Methods:**

- “inserted in the review” → “reviewed”

**Data Extraction:**

- “everyone” → “both”

Is the study design appropriate for the research question?

Partly

Is the rationale for, and objectives of, the study clearly described?

Yes

Are sufficient details of the methods provided to allow replication by others?

Yes

Are the datasets clearly presented in a useable and accessible format?

Yes

Reviewer Expertise:

Paediatric Respiratory disease

I confirm that I have read this submission and believe that I have an appropriate level of expertise to confirm that it is of an acceptable scientific standard, however I have significant reservations, as outlined above.

# Response

**Published:** 2024-05-29

**Authors:** danilo buonsenso

thank you very much, we changed as you suggested
